# Preparation and Gas-Sensitive Properties of SnO_2_@Bi_2_O_3_ Core-Shell Heterojunction Structure

**DOI:** 10.3390/nano15020129

**Published:** 2025-01-16

**Authors:** Jin Liu, Yixin Gao, Yuanyuan Lv, Mengdi Yang, Haoru Guo, Neng Li, Danyang Bai, Anyi Wang

**Affiliations:** School of Communication and Information Engineering, Xi’an University of Science and Technology, Xi’an 710054, China

**Keywords:** SnO_2_@Bi_2_O_3_ heterojunctions, hydrothermal method, gas sensors, ethanol

## Abstract

The SnO_2_@Bi_2_O_3_ core-shell heterojunction structure was designed and synthesized via a hydrothermal method, and the structure and morphology of the synthesized samples were characterized using X-ray diffraction (XRD), scanning electron microscopy (SEM), and X-ray photoelectron spectroscopy (XPS). Based on the conclusions from XRD and SEM, it can be observed that as the hydrothermal temperature increases, the content of Bi_2_O_3_ coated on the surface of SnO_2_ spheres gradually increases, and the diameter of Bi_2_O_3_ nanoparticles also increases. At a hydrothermal temperature of 160 °C, the SnO_2_ spheres are fully coated with Bi_2_O_3_ nanoparticles. This paper investigated the gas-sensitive performance of the SnO_2_@Bi_2_O_3_ sensor towards ethanol gas. Gas sensitivity tests at the optimal operating temperature of 300 °C showed that the composite prepared at 160 °C achieved a response value of 19.7 for 100 ppm ethanol. Additionally, the composite exhibited excellent response to 100 ppm ethanol, with a response time of only 4 s, as well as good repeatability. The excellent gas-sensitive performance of the SnO_2_@Bi_2_O_3_ core-shell heterojunction towards ethanol gas is attributed to its p-n heterojunction material properties. Its successful preparation contributes to the realization of high-performance heterostructure ethanol gas sensors.

## 1. Introduction

With the rapid development of industry, environmental pollution has become increasingly severe. Large quantities of untreated gasses containing harmful substances are being released into the atmosphere, posing significant threats to human health [1]. As a result, gas detection has become a key area of focus. Gas sensors, which play a vital role in environmental monitoring and air quality detection, have attracted growing research interest. Among them, metal oxide semiconductors (MOS) have garnered significant attention as gas-sensitive materials due to their simple structure, ease of fabrication, and real-time monitoring capabilities [2,3].

Tin dioxide (SnO_2_) is a common MOS material and is widely used in gas sensing materials due to its excellent electrical conductivity, wide bandgap (E_g_ = 3.6 eV), tunable resistivity, and sensitivity and selectivity to various gasses [4,5,6]. However, pure SnO_2_ suffers from low response and poor selectivity, and its gas sensing performance has not yet reached the expected targets. Many researchers have employed composite structures to enhance the performance of gas sensors. The formation of heterostructures, composed of two or more semiconductor materials, has been explicitly proven to improve gas sensor performance [7,8,9,10,11]. For instance, SnO_2_@SnS_2_ heterojunctions exhibited excellent performance in NO_2_ detection [12]. Researchers synthesized NiO/SnO_2_ hollow spheres and constructed p-n heterojunctions, significantly enhancing the sensitivity to triethylamine [13]. J.H. Kim et al. demonstrated that Co_3_O_4_/SnO_2_ composite nanofibers exhibited significantly enhanced response to acetone gas at 350 °C compared to pure SnO_2_ [14]. Zhang et al. developed a novel gas sensor based on ZnO/SnO_2_, which showed a response to 2000 ppm ethanol vapor that was 7 times higher than that of the original ZnO sensor [15]. Furthermore, Chen et al. reported that the SnO_2_/TiO_2_ heterojunction sensor achieved a response value of 9.58 to 100 ppm ethanol, which was 1.88 times that of SnO_2_ nanoparticles [16]. This improvement is attributed to the synergistic effect of band structure modulation and the formation of heterojunctions between the two semiconductors, which increases the electron depletion layer and improves charge carrier separation.

Bismuth oxide (Bi_2_O_3_) is another common MOS material with a bandgap of 2.79 eV. Due to its high refractive index and high dielectric constant, it has promising application prospects [17]. It is often combined with other metal oxides for constructing gas sensors [18,19,20,21]. For example, A. Montenegro et al. synthesized SnO_2_-Bi_2_O_3_ composites via the polymer precursor method, demonstrating that the introduction of bismuth significantly enhanced the sensor’s response to oxygen [22]. Jae Hoon Bang et al. proposed a highly sensitive and selective NO_2_ sensor based on Bi_2_O_3_ -modified branched SnO_2_ nanowires (NWs) [23]. Additionally, Yang et al. incorporated Bi_2_O_3_ particles as external additives onto the surface of SnO_2_ nanoparticles (NPs) for the efficient detection of oxygenated volatile organic compounds (VOCs) [24]. Therefore, SnO_2_@Bi_2_O_3_ composite materials exhibit potential for achieving reliable gas sensors, warranting further investigation in this field.

In this paper, SnO_2_@Bi_2_O_3_ core-shell heterojunctions were prepared at different temperatures using a hydrothermal method. The crystal structure, microstructure, and chemical states of the materials were characterized. The gas-sensing performance was tested to determine the optimal operating temperature of the sensor. The response and recovery times of the sensor were calculated, and the transient current curves of the sensors exposed to 5–100 ppm ethanol were analyzed. The effect of sensor repeatability was explored. This paper focuses on analyzing the gas-sensing mechanism of the sensors and reveals that the improvement in ethanol gas-sensing performance of SnO_2_@Bi_2_O_3_ composites primarily originates from the p-n heterojunction.

## 2. Experimental Section

### 2.1. Synthesis of SnO_2_

First, 8.098 g of SnCl_4_·5H_2_O (Beijing Tianyun Chemical Reagent Co., Ltd., Beijing, China) and 0.2 g of PVP (polyvinylpyrrolidone) (Sinopharm Chemical Reagent Co., Ltd., Shanghai, China) were dissolved in 35 mL of deionized water and stirred for 30 min until fully mixed. Subsequently, 6.02 g of NaOH(Sichuan Xilong Science Co., Ltd., Chengdu, China) was dissolved in 35 mL of deionized water and stirred at room temperature for 30 min to form a colorless and transparent NaOH solution. The NaOH solution was added dropwise to the SnCl_4_·5H_2_O solution, and the mixture was stirred for 30 min to form a transparent and uniform precursor solution. The precursor solution was transferred into a polytetrafluoroethylene reactor and reacted at 180 °C for 10 h. After natural cooling to room temperature, the sample was collected and washed five times with deionized water and anhydrous ethanol until neutral. Finally, the sample was dried at 60 °C for 12 h to obtain the final SnO_2_ sample.

### 2.2. Synthesis of SnO_2_@Bi_2_O_3_

First, 0.679 g of Bi(NO_3_)_3_·5H_2_O (Sinopharm Chemical Reagent Co., Ltd., Shanghai, China) and 0.168 g of NaOH were dissolved separately in 35 mL of deionized water and stirred for 30 min to form uniform solutions. The NaOH solution was added dropwise to the Bi(NO_3_)_3_·5H_2_O solution, forming a stable solution. Subsequently, 0.8 g of SnO_2_ prepared in the first step was added, and the mixture was stirred for another 30 min. The resulting solution was transferred into a polytetrafluoroethylene reactor and reacted at different temperatures (100 °C, 120 °C, 140 °C, and 160 °C) for 4 h. After natural cooling to room temperature, the samples were collected and washed five times with deionized water and anhydrous ethanol until neutral. Finally, the sample was dried at 70 °C for 12 h to obtain the final samples.

### 2.3. Material Characterization

The crystal structure of the samples was measured using an X-ray diffractometer (XRD, Bruker D8 Advance, Bruker Corporation, Karlsruhe, Germany). The microscopic morphology and element content of the prepared samples were analyzed using a scanning electron microscope (SEM, GeminiSEM 360, Carl Zeiss AG, Oberkochen, Germany) with energy-dispersive X-ray spectrum (EDS). X-ray photoelectron spectroscopy (XPS, Thermo Scientific-ESCALAB Xi+, Thermo Fisher Scientific, Waltham, MA, USA) was used to determine the surface composition and chemical states of the elements.

### 2.4. Sensor Fabrication and Gas Sensing

The fabrication process of the gas sensor is as follows: the prepared sample was mixed with an appropriate amount of deionized water and grinded to form a slurry. The slurry was then coated onto a clean ceramic tube equipped with a pair of gold electrodes to serve as the testing electrode. The ceramic tube was sintered in air at 300 °C for 3 h to enhance the material’s stability. Finally, a nickel–chromium heating wire was inserted and welded onto the hexagonal base of the ceramic tube, forming an indirectly heated gas sensor.

The sensor response was defined as $I_{g}∕I_{a}$, where $I_{a}$ represents the sensor current in air and $I_{g}$ represents the current in the target gas.

## 3. Results and Discussion

### 3.1. Characterization of the SnO_2_@Bi_2_O_3_

**Figure 1:**
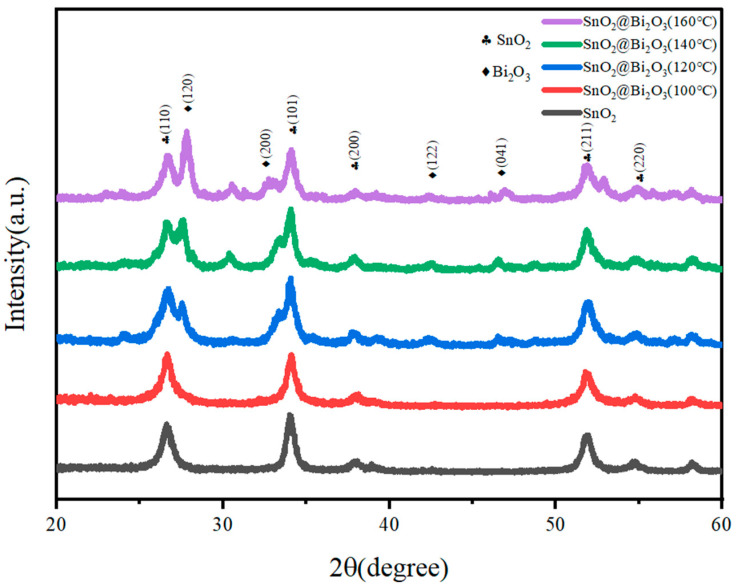
XRD spectra of SnO_2_, SnO_2_@Bi_2_O_3_ (100 °C), SnO_2_@Bi_2_O_3_ (120 °C), SnO_2_@Bi_2_O_3_ (140 °C), and SnO_2_@Bi_2_O_3_ (160 °C).

The crystal structures of SnO_2_, SnO_2_@Bi_2_O_3_ (100 °C), SnO_2_@Bi_2_O_3_ (120 °C), SnO_2_@Bi_2_O_3_ (140 °C), and SnO_2_@Bi_2_O_3_ (160 °C) samples were analyzed by XRD, and the results are presented in Figure 1. As shown in the figure, the XRD peaks of the synthesized SnO_2_ powder were observed at 2θ = 26.709°, 34.294°, 37.911°, and 52.03°, corresponding to the (110), (101), (200), and (211) planes of the tetragonal phase SnO_2_ (JCPDS No. 41-1445). This indicates that the SnO_2_ synthesized by the hydrothermal method possesses high purity and good crystallinity. For the SnO_2_@Bi_2_O_3_ composite material, the diffraction peaks not only include those of tetragonal SnO_2_ but also exhibit additional peaks at 2θ = 27.526°, 33.419°, and 46.474°, which correspond to the (120), (200), and (122) planes of Bi_2_O_3_ (JCPDS No. 41-1449). This confirms that the prepared material is indeed SnO_2_@Bi_2_O_3_. Moreover, the intensity of the Bi_2_O_3_ diffraction peaks increased with the reaction temperature, indicating improved crystallinity or higher Bi_2_O_3_ content in the SnO_2_@Bi_2_O_3_ composite.

**Figure 2:**
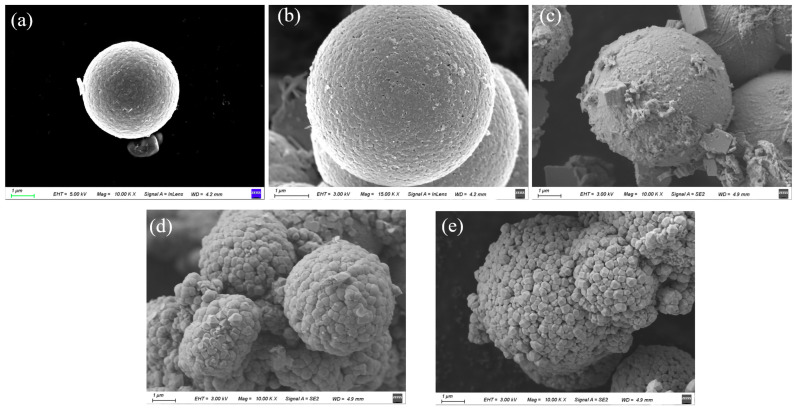
SEM images of (**a**) SnO_2_, (**b**) SnO_2_@Bi_2_O_3_ (100 °C), (**c**) SnO_2_@Bi_2_O_3_ (120 °C), (**d**) SnO_2_@Bi_2_O_3_ (140 °C), and (**e**) SnO_2_@Bi_2_O_3_ (160 °C).

The morphology and microstructure of the SnO_2_@Bi_2_O_3_ composite materials were further investigated using SEM. As shown in Figure 2a, pure SnO_2_ exhibits a typical spherical structure with a rough surface and an average diameter of approximately 4 µm. Figure 2b shows the SnO_2_@Bi_2_O_3_ composite synthesized at 100 °C, where a small amount of Bi_2_O_3_ is sparsely distributed on the SnO_2_ surface. This could be attributed to the fact that at low temperatures, the Bi_2_O_3_ grains are relatively small and not fully crystallized, existing predominantly in an amorphous or poorly crystallized state. As the temperature increased to 120 °C, Bi_2_O_3_ gradually formed sheet-like and flocculent structures, partially covering the SnO_2_ surface (Figure 2c). The increase in temperature promotes further crystallization of Bi_2_O_3_ and may lead to the formation of two-dimensional structures, such as sheet-like shapes, through self-assembly. The formation of these sheet-like structures can be attributed to the differences in crystal surface energy and growth rates, causing Bi_2_O_3_ to preferentially grow along specific crystal planes, resulting in sheet-like or flocculent morphologies. When the temperature was further elevated to 140 °C (Figure 2d), the morphology of Bi_2_O_3_ underwent a significant change, with the sheet-like and flocculent structures disappearing and being replaced by well-defined polyhedral structures. The SnO_2_surface was almost completely covered by Bi_2_O_3_, with the coverage becoming more uniform, though some residual sheet-like structures remained. At 160 °C (Figure 2e), the sheet-like Bi_2_O_3_ structures completely disappeared, and the surface morphology transformed into uniform polyhedron, resulting in a fully encapsulated structure, consistent with the XRD results. This transformation may be related to the rearrangement of crystals, crystal plane growth, and self-assembly mechanisms at high temperatures. At elevated temperatures, the solubility and diffusion rate of Bi_2_O_3_ increase, facilitating the crystal growth and optimized arrangement of Bi_2_O_3_ grains, driving the transition from sheet-like structures to polyhedral morphologies. The interfacial interaction between the SnO_2_ surface and Bi_2_O_3_ also promotes the uniform coating of Bi_2_O_3_, thereby forming stable polyhedral nanoparticles.

**Figure 3:**
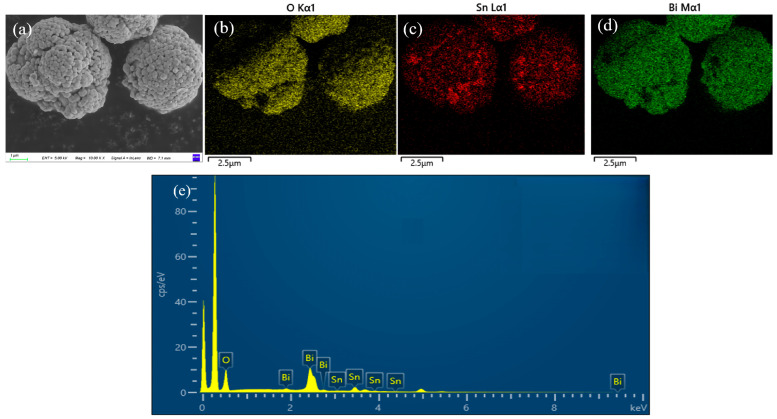
(**a**) SEM image of SnO_2_@Bi_2_O_3_ (160 °C); (**b**–**d**) Elemental mapping images of O, Sn, and Bi, respectively; (**e**) EDS of SnO_2_@Bi_2_O_3_ (160 °C).

EDS mapping of the SnO_2_@Bi_2_O_3_ sample prepared at 160 °C was performed to determine the distribution and content of elements on the sample surface. As shown in Figure 3b–d, oxygen (O, yellow), tin (Sn, red), and bismuth (Bi, green) are uniformly distributed across the sample surface. Further quantitative analysis of the elemental composition (Figure 3e and Table 1) revealed that the sample contains 79.3% O, 14.6% Bi, and 6.1% Sn. Additionally, no other elements were detected, indicating that the prepared sample is composed of SnO_2_@Bi_2_O_3_ composite material, which is consistent with the results of XRD and SEM analyses.

**Figure 4:**
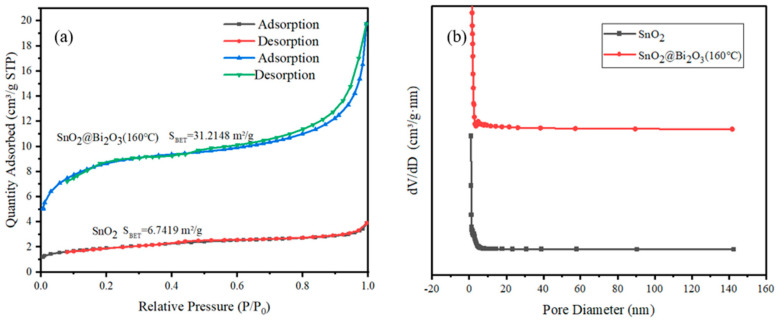
Nitrogen adsorption–desorption isotherms (**a**) and pore size distribution curves (**b**) for pure SnO_2_ and SnO_2_@Bi_2_O_3_ (160 °C).

The structure and specific surface area of materials are key factors affecting their gas sensitivity performance. Increasing the contact area between gas-sensitive materials and gasses can enhance the oxygen adsorption capacity on the material surface. In this study, both the SnO_2_@Bi_2_O_3_ (160 °C) composite material and pure SnO_2_ were tested using BET and BJH methods. As shown in Figure 4a, the adsorption–desorption isotherms of the SnO_2_@Bi_2_O_3_ (160 °C) composite material can be classified as Type IV, while those of SnO_2_ can be classified as Type II. Figure 4b shows the pore size distribution curves for both SnO_2_@Bi_2_O_3_ (160 °C) and pure SnO_2_, with the main peaks occurring at 2–4 nm. Table 2 presents the BET data for both SnO_2_@Bi_2_O_3_ (160 °C) and SnO_2_, where the specific surface area of SnO_2_@Bi_2_O_3_ (160 °C) is 31.2148 m^2^/g, approximately 5 times that of the pure SnO_2_ nanospheres (6.7419 m^2^/g). This indicates that the SnO_2_@Bi_2_O_3_ composite material prepared in this study has a larger specific surface area, enhancing the rate of electron exchange between ethanol gas and the semiconductor material, thus creating favorable conditions for the adsorption, diffusion, and reaction of the test gas. The average pore diameter of SnO_2_@Bi_2_O_3_ (160 °C) is about 2.9790 nm, slightly larger than that of SnO_2_ at 2.8669 nm. The pore volume of SnO_2_@Bi_2_O_3_ (160 °C) is 0.020202 cm^3^/g, significantly greater than that of SnO_2_ at 0.004116 cm^3^/g, corresponding to its higher specific surface area, suggesting that the material provides more pore space for gas adsorption.

**Figure 5:**
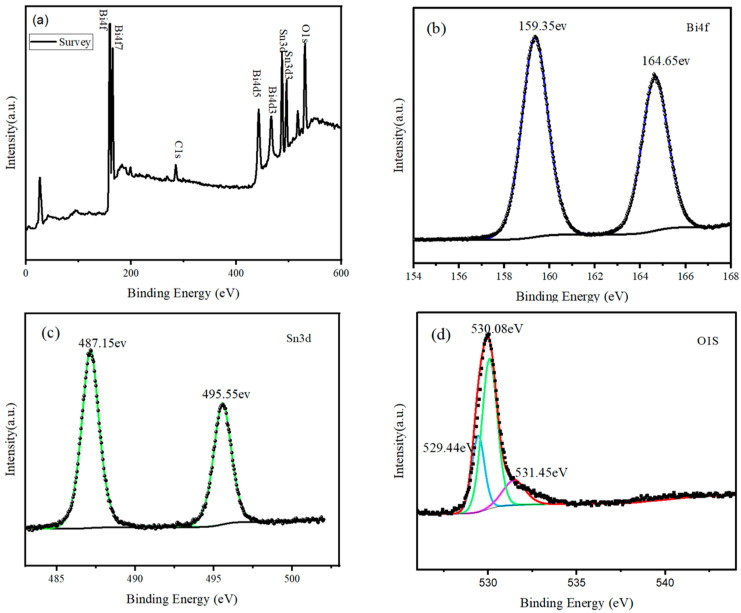
XPS spectra of SnO_2_@Bi_2_O_3_ (160 °C): (**a**) Survey spectrum, (**b**) Bi 4f spectrum, (**c**) Sn 3d spectrum, and (**d**) O 1s spectrum.

The chemical states and surface composition of the SnO_2_@Bi_2_O_3_ composite prepared at 160 °C were analyzed using XPS, as shown in Figure 5. The survey spectrum (Figure 5a) reveals distinct peaks corresponding to Sn, O, and Bi elements, confirming their presence in the sample. The signal from C is attributed to calibration, with the C 1s peak at 284.8 eV used as the reference for calibration. Figure 5b shows the high-resolution Bi 4f spectrum, with peaks at binding energies of 159.35 eV and 164.65 eV, corresponding to Bi 4f_7_/_2_ and Bi 4f_5_/_2_, respectively, which are characteristic peaks of Bi^3+^ [25,26]. The high-resolution Sn 3d spectrum (Figure 5c) exhibits peaks at 487.15 eV and 495.55 eV, corresponding to Sn 3d_5_/_2_ and Sn 3d_3_/_2_, indicating that Sn exists in the form of Sn^4+^ in the composite [27,28]. Figure 5d presents the high-resolution O 1s spectrum, which can be divided into three sub-peaks with binding energies at 529.44 eV, 530.08 eV, and 531.45 eV, corresponding to oxygen in Bi_2_O_3_ (blue), oxygen in SnO_2_ (green), and adsorbed oxygen (purple), respectively [29,30]. The XPS results further confirm that the SnO_2_@Bi_2_O_3_ composite consists of SnO_2_ and Bi_2_O_3_.

### 3.2. Gas-Sensing Performances

**Figure 6:**
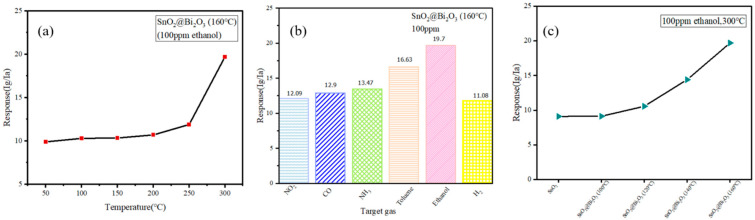
(**a**) Response of SnO_2_@Bi_2_O_3_ (160 °C) to 100 ppm ethanol at different operating temperatures; (**b**) Selectivity of SnO_2_@Bi_2_O_3_ (160 °C) at 300 °C for various gasses; (**c**) Response of SnO_2,_ SnO_2_@Bi_2_O_3_ (100 °C), SnO_2_@Bi_2_O_3_ (120 °C), SnO_2_@Bi_2_O_3_ (140 °C), and SnO_2_@Bi_2_O_3_ (160 °C) to 100 ppm ethanol at 300 °C.

Metal oxide semiconductors require sufficient operating temperatures to activate the surface adsorption of oxygen and promote chemical reactions. Thus, the optimum operating temperature needs to be determined [31,32,33]. As shown in Figure 6a, the response of the SnO_2_@Bi_2_O_3_ sensor prepared at 160 °C to 100 ppm ethanol was tested over a temperature range of 50 °C to 300 °C. The response value increased with rising temperature and reached a maximum of 19.7 at 300 °C. This indicates that the optimum operating temperature of the SnO_2_@Bi_2_O_3_ (160 °C) sensor is 300 °C. High selectivity is a critical requirement for gas sensors to avoid interference from other gasses during the detection process. Thus, the selectivity of the SnO_2_@Bi_2_O_3_ (160 °C) sensor was tested at 300 °C against various gasses (including NO_2_, NH_3_, toluene, H_2_, and CO) at a concentration of 100 ppm, as shown in Figure 6b. The response values for NO_2_, CO, NH_3_, toluene, and H_2_ were 12.09, 12.9, 13.47, 16.63, and 11.08, respectively, which were all lower than the response value of 19.7 for ethanol. This demonstrates the excellent selectivity of the SnO_2_@Bi_2_O_3_ (160 °C) sensor. At the optimal operating temperature of 300 °C, the responses of SnO_2_ and SnO_2_@Bi_2_O_3_ composites prepared at different temperatures to 100 ppm ethanol gas were compared, as shown in Figure 6c. It can be observed that SnO_2_ exhibits the lowest response value, while SnO_2_@Bi_2_O_3_ (160 °C) shows the highest response value. According to Table 3, it is evident that the response value of SnO_2_ is approximately 9, whereas the response values of SnO_2_@Bi_2_O_3_ (100 °C), SnO_2_@Bi_2_O_3_ (120 °C), and SnO_2_@Bi_2_O_3_ (140 °C) were 9, 10.5, and 13.6, respectively. The highest response value of 19.7 was observed for SnO_2_@Bi_2_O_3_ (160 °C), significantly outperforming the other samples.

**Figure 7:**
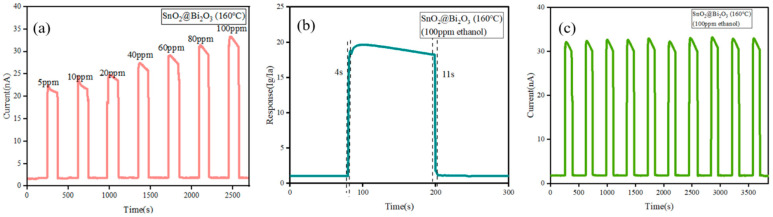
(**a**) Transient current curves of the SnO_2_@Bi_2_O_3_ (160 °C) sensor to different concentrations of ethanol at 300 °C; (**b**) Response and recovery curves of the SnO_2_@Bi_2_O_3_ (160 °C) sensor to 100 ppm ethanol at 300 °C; (**c**) Transient current curves of the SnO_2_@Bi_2_O_3_ (160 °C) sensor over ten cycles for 100 ppm ethanol at 300 °C.

As shown in Figure 7a, the transient current curves of the SnO_2_@ Bi_2_O_3_ sensor prepared at 160 °C to ethanol gas at 300 °C indicate gas concentrations ranging from 5 ppm to 100 ppm. It can be observed from the figure that as the ethanol gas concentration increases, the current of the SnO_2_@Bi_2_O_3_ (160 °C) sensor shows a continuous upward trend, reaching a maximum value of approximately 32 nA at 100 ppm ethanol gas. The response and recovery characteristics of the SnO_2_@Bi_2_O_3_ (160 °C) sensor to 100 ppm ethanol at 300 °C are shown in Figure 7b. The response and recovery times were 4 s and 11 s, respectively, demonstrating the sensor’s fast response and recovery capabilities. Figure 7c presents the transient current curves of the SnO_2_@Bi_2_O_3_ (160 °C) sensor over ten cycles for 100 ppm ethanol at 300 °C. The analysis indicates that the current of the SnO_2_@Bi_2_O_3_ (160 °C) sensor remained stable over time, with negligible fluctuations, consistently maintaining a value of approximately 32 nA. This result demonstrates the excellent repeatability of the sensor.

**Figure 8:**
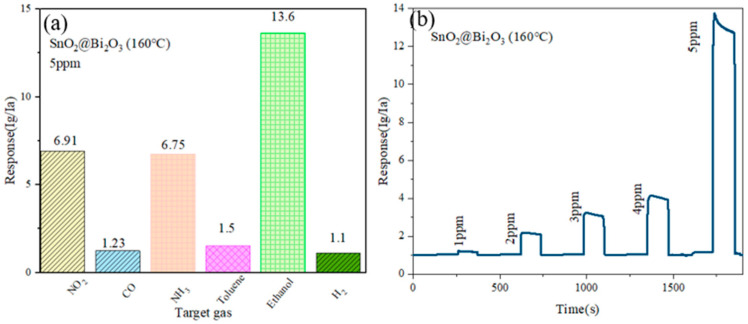
(**a**) SnO_2_@Bi_2_O_3_ (160 °C) sensor response to 5 ppm of different gasses; (**b**) Response of SnO_2_@Bi_2_O_3_ (160 °C) to 1–5 ppm concentration of ethanol.

Figure 8a shows the response of the SnO_2_@Bi_2_O_3_ (160 °C) sensor to the target gas (ethanol) and interfering gasses (NO_2_, NH_3_, toluene, H_2_, and CO) at a concentration of 5 ppm. The decision to test these gasses at low concentrations is based on the fact that ethanol is typically derived from chemical production, while other gasses (NO_2_, NH_3_, toluene, H_2_, and CO) are often produced by industrial emissions. These gasses may coexist in the environment, and ethanol leaks in the environment generally occur at low concentrations. However, when mixed with other gasses, the accuracy of detection could be affected. Therefore, the sensor’s response to these gasses was tested at a low concentration of 5 ppm.

At 300 °C, the SnO_2_@Bi_2_O_3_ (160 °C) sensor showed a response value of 13.6 to 5 ppm ethanol gas, while its response values to other interfering gasses (NO_2_, NH_3_, toluene, H_2_, and CO) at the same concentration were 6.91, 6.75, 1.5, 1.1, and 1.23, respectively. It can be observed that the sensor’s response to the target gas ethanol is significantly higher than its response to the interfering gasses. This indicates that even under low concentration conditions, the SnO_2_@Bi_2_O_3_ (160 °C) sensor can effectively detect ethanol gas. Figure 8b shows the test results of the SnO_2_@Bi_2_O_3_ (160 °C) sensor within the ethanol concentration range of 1–5 ppm. It can be observed that the sensor has no response below 1 ppm, but begins to respond at 1 ppm, with a response value of 1.21, indicating a detection limit of 1 ppm. According to Table 4, the response values remain stable at approximately 1.21 in the range of 1 ppm to 1.4 ppm but increase significantly to 1.562 at 1.5 ppm, demonstrating a resolution of 0.5 ppm for the SnO_2_@Bi_2_O_3_ (160 °C) sensor.

### 3.3. Gas Sensing Mechanism

SnO_2_ is a typical n-type semiconductor metal oxide that follows the gas sensing mechanism of n-type semiconductors. The adsorption and desorption of target gas molecules on the surface of SnO_2_ cause changes in its resistance [34]. When the SnO_2_ sensor is exposed to air at its optimal operating temperature, a large amount of oxygen is adsorbed onto the material’s surface. At this point, some conduction band electrons transfer to the adsorbed oxygen, existing in the form of O^−^, forming a thick depletion layer [35,36,37,38]. As a result, the potential barrier is higher, the resistance increases, and the current decreases. The specific reaction process is as follows:

(1) $$O_{2(gas)}\to O_{2(ads)}$$

(2) $$O_{2(ads)}+e^{-}\to O_{2(ads)}^{-},T<100{}^{\circ}C$$

(3) $$O_{2\left(ads\right)}^{-}+e^{-}\to {2O}^{-},100{}^{\circ}C\leq T\leq 300{}^{\circ}C$$

**Figure 9:**
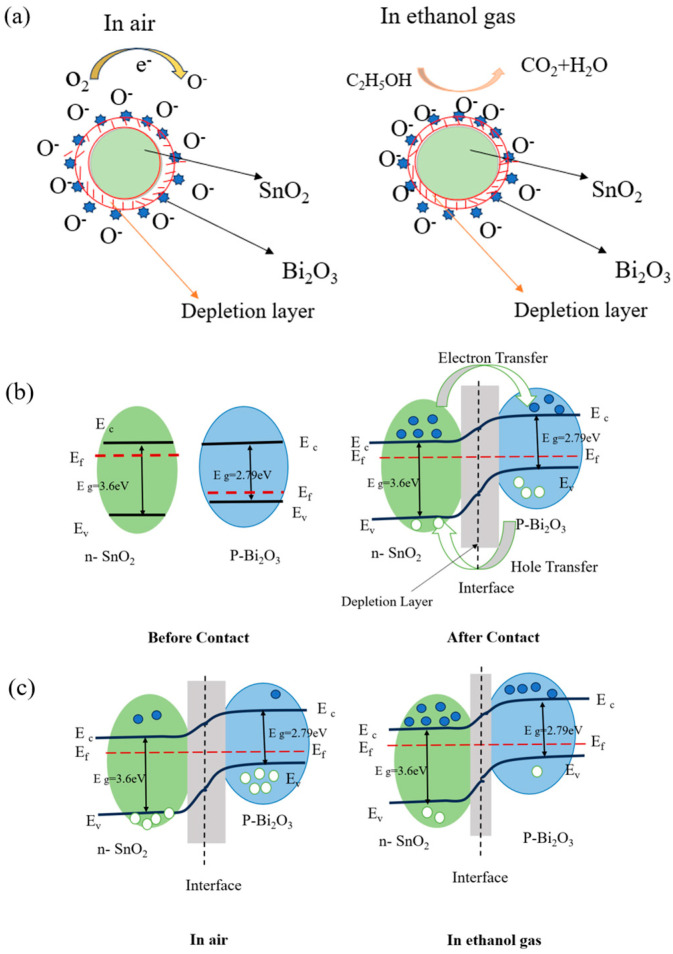
(**a**) Schematic diagram of the sensing mechanism of the SnO_2_@Bi_2_O_3_ core-shell heterojunction when exposed to air and ethanol; (**b**) Band diagrams of SnO_2_ and Bi_2_O_3_ before and after contact; (**c**) Band diagram of the SnO_2_@Bi_2_O_3_ core-shell heterojunction in air and ethanol gas.

When the SnO_2_ sensor comes into contact with ethanol (C_2_H_5_OH) gas, the adsorbed oxygen on the surface reacts with C_2_H_5_OH to produce CO_2_ and H_2_O, as shown in Figure 9a. In this process, electrons return to the conduction band of the sensing material, thinning the depletion layer, thereby reducing the sensor’s resistance and increasing the current. The reaction equation is as follows:

(4) $$C_{2}H_{5}OH+6O^{-}\to 2{CO}_{2}+3H_{2}O+6e^{-}$$

When the sensor is returned to normal air from the tested gas, oxygen re-adsorbs onto the surface of SnO_2_, and the conduction band electrons exist again in the form of O^−^, causing the resistance to increase and the current to decrease.

The enhanced sensitivity of the SnO_2_@Bi_2_O_3_ composite material to ethanol gas may be attributed to the heterojunction effect [39,40,41]. Based on the grain boundary potential barrier model, Figure 9b shows that SnO_2_ and Bi_2_O_3_ materials have different Fermi energy levels. When SnO_2_ is combined with Bi_2_O_3_ to form an n-p heterojunction, electrons flow from the Fermi level of SnO_2_ (which is higher) to the Fermi level of Bi_2_O_3_ (which is lower) until a dynamic equilibrium is reached. At this point, the energy bands of SnO_2_ and Bi_2_O_3_ bend in the space charge region of the n-p junction, forming a potential barrier. Figure 9c illustrates that when the SnO_2_@Bi_2_O_3_ heterostructure is exposed to reducing ethanol gas, ethanol reacts with the oxygen adsorbed (O^−^) on the surface of SnO_2_, releasing a large number of electrons. These electrons are injected into the conduction band of SnO_2_, lowering its resistance. Simultaneously, some electrons diffuse to the p-type Bi_2_O_3_ interface, causing electron–hole recombination and reducing the hole concentration in Bi_2_O_3_, which further decreases the thickness of the depletion layer and the height of the interface potential barrier. Moreover, ethanol gas may also directly react with the holes on the surface of Bi_2_O_3_, releasing more electrons. These electrons are transferred to the conduction band of SnO_2_, further reducing the overall resistance of the composite material and increasing the current. Ultimately, the synergistic effects of these processes significantly enhance the sensitivity of the SnO_2_@Bi_2_O_3_ heterojunction material to ethanol gas [42,43,44,45].

## 4. Conclusions

In summary, this study successfully prepared SnO_2_@Bi_2_O_3_ heterostructures via a simple hydrothermal method and evaluated their gas-sensing performance. As the hydrothermal temperature increases, the content of Bi_2_O_3_ coated on the surface of SnO_2_ spheres gradually increases, and the diameter of the Bi_2_O_3_ nanoparticles also increases. When the hydrothermal temperature reaches 160 °C, the SnO_2_ spheres are completely coated with Bi_2_O_3_ nanoparticles. The test results demonstrated that the SnO_2_@Bi_2_O_3_ heterostructure exhibited excellent sensitivity to ethanol gas. At 300 °C, the response value of SnO_2_@Bi_2_O_3_ (160 °C) to 100 ppm ethanol reached 19.7, with response and recovery times of 4 s and 11 s, respectively. The composite material also showed excellent repeatability. Based on the characterization results, the performance enhancement was attributed to the presence of p-n heterojunctions on the material surface. Therefore, the SnO_2_@Bi_2_O_3_ heterostructure provides a promising strategy for ethanol gas detection and sensor development.

## Figures and Tables

**Table 1:** Elemental composition of the SnO_2_@Bi_2_O_3_ (160 °C) sample.

Element	Atomic %
O	79.3
Sn	6.1
Bi	14.6
Total	100

**Table 2:** The BET specific surface areas of different samples.

Samples	S_BET_ (m^2^/g)	Average Pore Diameter (nm)	Pore Volume (cm^3^/g)
SnO_2_	6.7419	2.8669	0.004116
SnO_2_@Bi_2_O_3_ (160 °C)	31.2148	2.9790	0.020202

**Table 3:** Response of SnO_2_, SnO_2_@Bi_2_O_3_ prepared at different temperatures to 100 ppm ethanol at 300 °C.

Ethanol Gas (100 ppm)	Sensor Response
SnO_2_	9
SnO_2_@Bi_2_O_3_ (100 °C)	9
SnO_2_@Bi_2_O_3_ (120 °C)	10.5
SnO_2_@Bi_2_O_3_ (140 °C)	13.6
SnO_2_@Bi_2_O_3_ (160 °C)	19.7

**Table 4:** Response of SnO_2_@Bi_2_O_3_ (160 °C) sensor to different concentrations of ethanol gas.

Concentration of Ethanol (ppm)	Sensor Response
1	1.21
1.1	1.21
1.2	1.21
1.3	1.21
1.4	1.21
1.5	1.562

## Data Availability

The data provided in this study are available from the corresponding author.
